# Differential regulation of TNFα and IL-6 expression contributes to immune evasion in prostate cancer

**DOI:** 10.1186/s12967-022-03731-x

**Published:** 2022-11-12

**Authors:** Ida Deichaite, Timothy J. Sears, Leisa Sutton, Daniel Rebibo, Kylie Morgan, Tyler Nelson, Brent Rose, Pablo Tamayo, Napoleone Ferrara, Fotis Asimakopoulos, Hannah Carter

**Affiliations:** 1 Department of Radiation Medicine and Applied Sciences, University of California San Diego, La Jolla, CA USA; 2 Moores Cancer Center, University of California San Diego, La Jolla, CA USA; 3 Bioinformatics and Systems Biology Program, University of California San Diego, La Jolla, CA USA; 4 VA San Diego Healthcare System, San Diego, CA USA; 5 Department of Urology, University of California San Diego, La Jolla, CA USA; 6 Division of Medical Genetics, Department of Medicine, University of California San Diego, La Jolla, CA USA; 7 Center for Novel Therapeutics, University of California San Diego, La Jolla, CA USA; 8 Department of Pathology, University of California San Diego, La Jolla, CA USA; 9 Division of Blood and Marrow Transplantation, Department of Medicine, University of California San Diego, La Jolla, CA USA

**Keywords:** Prostate cancer, TNF, IL-6, ADAMTS-4, AP-1, FOSB, Bevacizumab, SELE, Macrophage polarization, Immune remodeling

## Abstract

**Background:**

The role of the inflammatory milieu in prostate cancer progression is not well understood. Differences in inflammatory signaling between localized and metastatic disease may point to opportunities for early intervention.

**Methods:**

We modeled PCa disease progression by analyzing RNA-seq of localized vs. metastatic patient samples, followed by CIBERSORTx to assess their immune cell populations. The VHA CDW registry of PCa patients was analyzed for anti-TNF clinical outcomes.

**Results:**

We observed statistically significant opposing patterns of IL-6 and TNFα expression between localized and metastatic disease. IL-6 was robustly expressed in localized disease and downregulated in metastatic disease. The reverse was observed with TNFα expression. Metastatic disease was also characterized by downregulation of adhesion molecule E-selectin, matrix metalloproteinase ADAMTS-4 and a shift to M2 macrophages whereas localized disease demonstrated a preponderance of M1 macrophages. Treatment with anti-TNF agents was associated with earlier stage disease at diagnosis.

**Conclusions:**

Our data points to clearly different inflammatory contexts between localized and metastatic prostate cancer. Primary localized disease demonstrates local inflammation and adaptive immunity, whereas metastases are characterized by immune cold microenvironments and a shift towards resolution of inflammation and tissue repair. Therapies that interfere with these inflammatory networks may offer opportunities for early intervention in monotherapy or in combination with immunotherapies and anti-angiogenic approaches.

## Background

Interleukin 6 (IL-6) in prostate cancer (PCa) is recognized as a potential mediator and biomarker of disease progression. Elevated IL-6 plasma levels have been implicated in PCa development and progression [1, 2]. Local production of IL-6 has been detected in androgen-independent PCa cell lines, arguing for its involvement in autocrine and paracrine functions [3, 4].

Both IL-6 and tumor necrosis factor alpha (TNFα) serum levels were shown to correlate with patient disease progression and survival, further establishing both cytokines as mediators and prognostic biomarkers [5]. However, the role of IL-6 in disease progression remains contested. An IL-6 antagonist (*siltuximab*) has been tested in clinical trials in PCa patients but had no clinical efficacy [6]. Other studies reported that in PCa patients, IL-6 is not detected in PCa cells apart from the stromal compartments [7]. It remains to be firmly established whether IL-6 is a driver or a surrogate biomarker of PCa progression.

To gain a better understanding of the role of pro-inflammatory cytokines in PCa progression, we analyzed messenger RNA (mRNA) levels of IL-6 and TNFα from 49 somatic tumor tissue samples (see “Methods” section). Somewhat contrary to published reports, we found that IL-6 expression decreased with disease progression as compared to localized tumors. However, TNFα expression levels increased through disease progression. Our IL-6 and TNFα expression data are in agreement with the results reported by Yu et al. [7] who examined the cellular origin of IL-6 and TNFα in PCa patients utilizing quantitative reverse transcription PCR (q-RT-PCR) as well as chromogenic in situ hybridization (CISM) studies. They reported that benign prostate tissue had higher expression of IL-6 mRNA than matched patient tumor samples while TNFα expression remained unchanged.

While there is cumulative evidence that both IL-6 and TNFα play an important role in inflammation and PCa progression, the regulatory pathways and the immune microenvironment associated with these cytokines are not well understood and deciphering their function will aid in developing new therapeutic options for patients.

## Methods

### NGS Data source and patient inclusion criteria

This was an institutional review board (IRB) approved retrospective cohort analysis performed on men with PCa (Protocol Number 190443). Eligible patients in our next generation sequencing (NGS) study had adequate primary tumor tissue sequenced using the CAP/CLIA validated Tempus xT test. Raw RNA-sequencing (RNA-seq) data from Tempus was analyzed.

### NGS Gene expression analysis and GSEA

Enrichment scores for the tumor samples were calculated using Single-sample Gene Set Enrichment Analysis (ssGSEA) projection [8]. Gene expression values were rank-normalized from their absolute expression, and an enrichment score within each tumor sample was calculated by evaluating the differences in the empirical cumulative distribution functions of the genes in the gene set relative to the remaining genes. A positive ssGSEA score indicates a significant overlap of the gene set with groups of genes at the top of the ranked list, while a negative ssGSEA score indicates a significant overlap of the gene set with groups of genes at the bottom of the ranked list. We used gene sets from the Molecular Signatures Database (MSigDB) [9–11]. To quantify the degree of association we used the Information Coefficient (IC) [12].

An empirical permutation test was used to compute p-values and assess statistical significance.

### NGS Custom signature matrix generation

Single cell RNA-seq (sc-RNA-seq) data was obtained from Chen et al. [13], which used 10X genomics sequencing technology to obtain reads from four metastatic and nine localized PCa samples. Using the Seurat single cell analysis package, these samples were arrayed as an expression matrix, normalized, then clustered on 2000 variable marker genes with a resolution setting of 1. Myeloid and T-cell populations were then identified via CD14 + and CD3D/E + [14] expression respectively. These subpopulations were similarly clustered into high resolution subpopulations and identified based on the following marker genes: CD4 Tregs (PMCH, FOXP3) [14, 15]; CD4 Memory Resting (TNFSF14, ATHL1) [14, 15]; Gamma Delta T Cell (DUSP2, CCL4, CD3D) [14, 15]; T Cell Follicular Helper (ICA1, PDCD1) [14, 15]; CD4 Naive (FLT3LG, ANKRD55) [14, 15]; CD4 Memory Activated (IFNG, CCL20) [14, 15]; CD8 T Cell (CD8A, CCL5, CD8B) [15]; NKT (GZMM, GNLY) [15]; B Cell Memory (GPR183) [15]; B Cell Naive (AIM2, GPR183) [15]; Mast cells (TRIB2, ZNF165, TPSAB1) [15]; M0 Macrophages (CYP27A1, ACP5) [15, 16]; M1 Macrophages [16, 17]; M2 Macrophages (TREM2, CD68, HLA-DQA1) [16, 17]; Monocytes (FCN1, S100A12, FPR1) [18]; Dendritic Cells Resting (CD1C, CD1E) [15]; and Dendritic Cells Activated (CCL22, LAMP3, IDO1) [15, 18].

A custom signature matrix was generated using these cell type gene expression profiles via the “Create Signature Matrix” function of CIBERSORTx [19]. This custom signature matrix of pre-metastatic and metastatic prostate immune cell populations was then applied to each of the RNA-seq cohorts to estimate immune cell infiltration via the “Impute Cell Fractions” module from CIBERSORTx.

### NGS Analysis of immune cell infiltration

R statistical software version 4.0.1 was used to measure the correlation between gene expression and immune cell infiltration approximations [20]. R^2^ values and p-values were calculated using the spearman correlation coefficient. Similarly, differences in immune cell infiltration approximations between pre-metastatic and metastatic populations were compared within cohorts and p-values were calculated using the Wilcoxon signed rank test.

### VHA Data source

Patient information for the clinical cohort was collected from the Veterans Health Administration (VHA) Corporate Data Warehouse (CDW), which contains health records of > 9 million veterans from approximately 170 VHA medical centers and 1000 outpatient sites [21]. This study was reviewed and approved by the VHA San Diego Healthcare System. Waivers of consent and authorization were granted by the Institutional Review Board and the Research and Development Committee of the VHA San Diego Healthcare System (Protocol Number 150169).

### VHA Study population

Patients in the VHA diagnosed with PCa from 2000 to 2014 were included in this cohort. Follow up ended on June 23, 2017. Patients with unknown initial treatment or clinical staging were excluded from the cohort.

### VHA Measures

We collected information on age at diagnosis, race, employment status, Gleason scores, T stage, and metastatic stage from the Veterans Affairs Informatics and Computing Infrastructure (VINCI) CDW Oncology Registry. Pre diagnostic prostate specific antigen (PSA) levels were collected from the VINCI Prostate Cancer Data Core to use as baseline nearest PSA level before diagnosis.

Outcomes of interest included associations of pre diagnostic treatment with any TNF antagonist use (*adalimumab, certolizumab, erelzi, golimumab, etanercept*) with PCa characteristics at diagnosis (Gleason score, clinical stage, and PSA), and long-term development of metastatic disease. Diagnosis of metastatic disease was obtained through the Prostate Cancer VINCI Data Core [22], which uses an internally developed Natural Language Processing (NLP) tool to identify cases of metastases in PCa patients. Time to event endpoints were calculated from the date of diagnosis to the event of interest or censored at the date of last follow up. Patients who died without experiencing an event were censored at the time of death in Cox proportional hazards models and counted as a competing event in cumulative incidence functions.

### VHA Statistical analysis

Univariable cumulative incidences of development of metastases were measured. Logistic regression models were used to measure associations between sociodemographic characteristics at time of diagnosis and TNF antagonist use. Age at diagnosis, African American ethnicity, and employment status at diagnosis were additional covariates for models with these outcomes: presenting with Gleason 8 or higher disease, presenting with T stage (3 or 4 vs. 1 or 2), presenting with a PSA > 20 ng/mL, and presenting with metastatic disease at diagnosis. Cox proportional hazards models controlling for the same variables were used to measure associations between pre diagnostic TNF antagonist use and development of metastases.

## Results

### PCa localized versus metastatic RNA-seq analysis

**Fig. 1:**
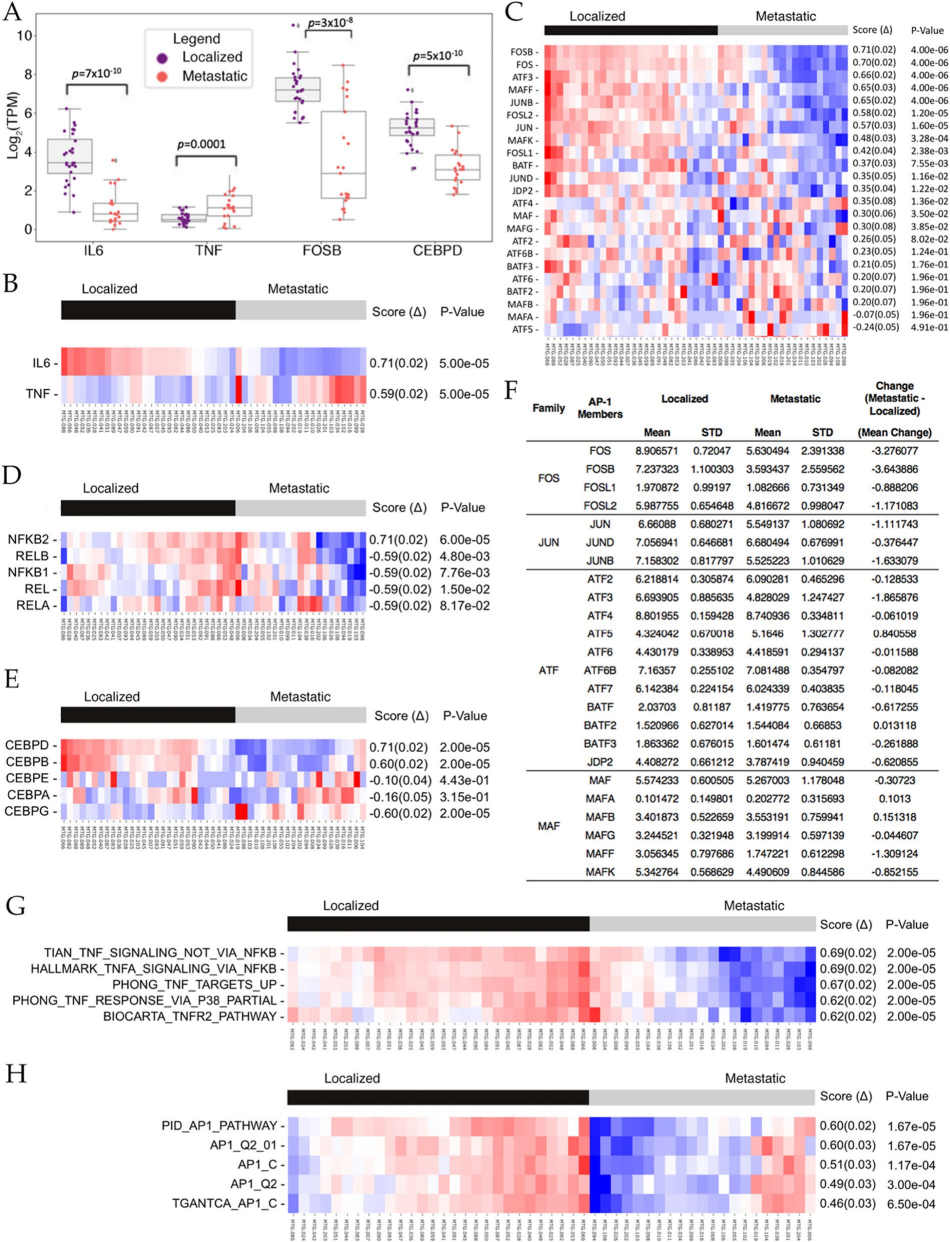
**A** Log_2_TPM expression of IL-6, TNFα, FOSB, and CEBPD in the PCa cohort split by localized and metastatic status. These genes were measured to have significantly differential expression between the localized and metastatic groups (two-tailed student’s t-test). Median and interquartile ranges are shown for each group. **B–E** heatmaps showing relative expression profiles (mRNA) of selected genes vs. localized/metastatic groupings of 49 PCa patients, where 28 were localized (black) and 21 were metastatic (grey). The genes shown are **B** TNFα and IL-6, **C.** AP-1 components **D** NF-kB and **E** CEBP family genes. On the right side of the heatmaps we show the Information Coefficient (Δ), a measure of association of each gene/gene set vs. the phenotype and the corresponding permutation-derived p-value. **F** mean values and standard deviations for all components of the AP-1 complex. **G**,** H** single-sample GSEA analysis heatmap showing the Information Coeficient (Δ) association between localized/metastatic groups and the expression of **G** NF-kB related gene sets and **H** AP-1 related gene sets. The 5 top scoring gene set are shown

We analyzed somatic tumor RNA-seq data for expression levels of TNFα and IL-6 in localized versus metastatic disease (Fig. 1). We observed elevated TNFα expression levels in metastatic disease (localized mean = 0.0557 vs. metastatic mean = 1.244 Log_2_TPM, p = 0.0001, Fig. 1A). IL-6 levels, in contrast, revealed a significant expression reduction in metastatic compared to localized disease (mean = 3.652 vs. mean = 1.101 log_2_TPM, p = 7 × 10^–10^, Fig. 1A). This result is demonstrated in a TNFα and IL-6 expression heatmap, as lower relative expression of IL-6 correlates with higher relative expression of TNFα, and vice versa, regardless of the inherent cohort heterogeneity (Fig. 1B).

We examined the upstream transcriptional factors (TFs) associated with TNFα and IL-6 regulation such as the Activator Protein 1 (AP-1) family of TF’s, NFkB and CEBP to decipher molecular mechanisms that may drive the differential expression of these cytokines in localized versus metastatic disease (Fig. 1C–F). We found that AP-1 TF FOSB had a particularly significant association with IL-6 expression (Fig. 1A, F). Other AP-1 TF’s such as FOS, ATF3, JUNB and CEBP binding protein CEBPD are also correlated with IL-6 expression but to a lesser extent than FOSB (Figs. 1A, C, F). The structure of AP-1 is that of a homo- or heterodimer composed of proteins representing FOS, JUN and ATF sub families [23, 24]. The dimerization specificity among various AP-1 TFs defines downstream target functional outcomes by binding to gene promoters that include IL-6, TNFα, SELE and many other genes [25–27]. Thus, the decrease of FOSB in metastatic disease may cause divergence between TNFα and IL-6 PCa expression levels by changing the available ratios of the corresponding translated proteins for dimerization.

**Fig. 2:**
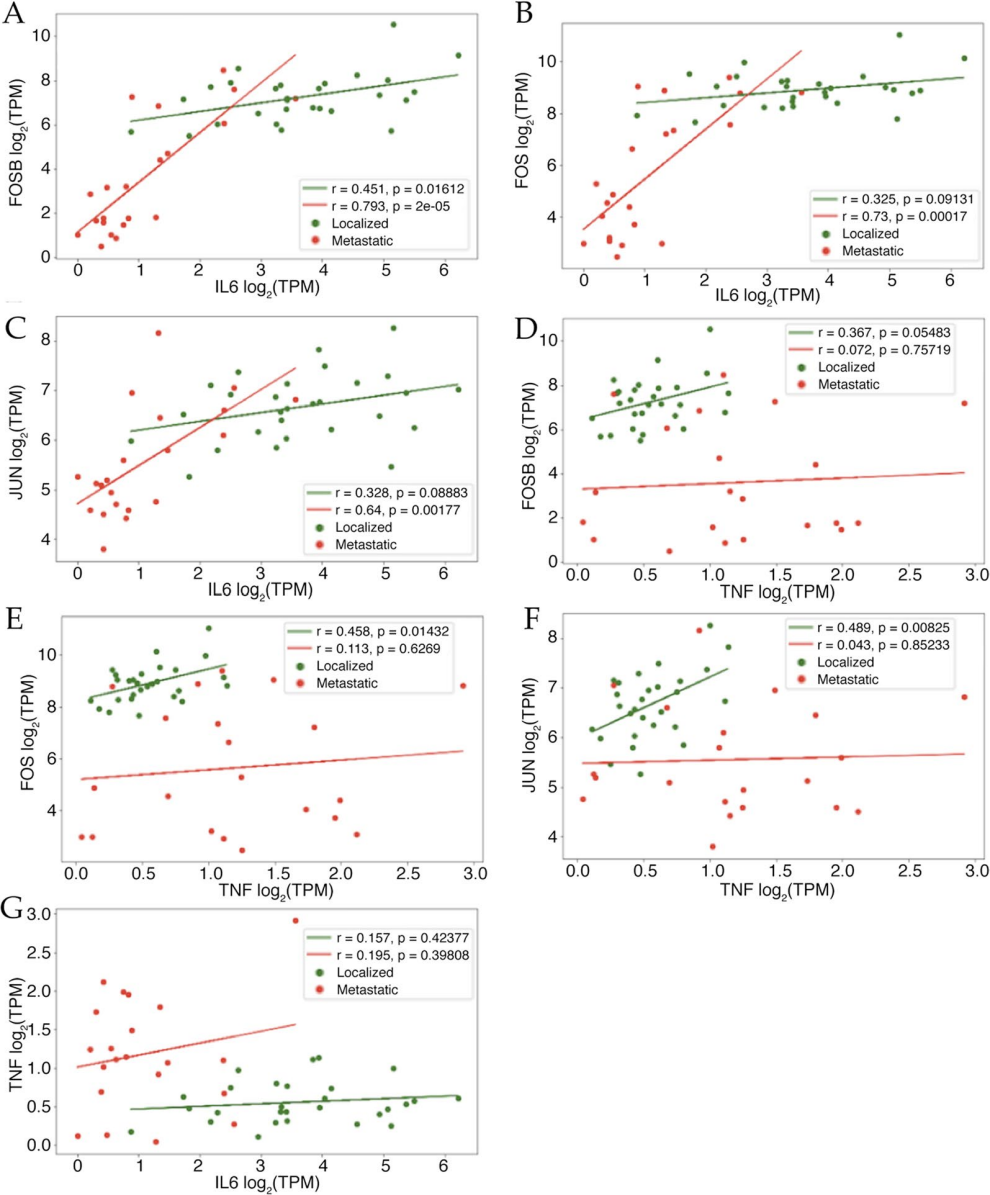
**A**–**G** Scatterplots with Pearson’s R calculated between AP-1 Complex subunit genes and TNFα/IL-6 log_2_TPM expression values from RNA-seq analysis of 49 PCa patients. Figure legend keys are as follow: “r”: Pearson’s R; “p”: P-Value; “Localized”: N = 28 localized patients; “Metastatic”: N = 21 metastatic patients; “TPM”: transcripts per million

To further explore the association between TNFα, IL-6 and AP-1 FOSB, FOS and JUN we performed standard correlation analysis and observed a significant linear correlation between IL-6 and FOSB (r = 0.79, Fig. 2A), IL-6 and FOS (r = 0.73, Fig. 2B) as well as IL-6 and JUN (r = 0.64, Fig. 2C) in the metastatic state. In localized disease FOSB displayed a modest linear correlation with IL-6 (r = 0.451, p = 0.016. Figure 2A). However, TNFα did not show significant association with FOSB, FOS or JUN in the metastatic setting (Fig. 2D–F). We observed a weaker linear correlation for all three AP-1 TFs with TNFα in localized disease (Fig. 2D–F). We also observed that TNFα and IL-6 are neither correlated significantly with each other in localized nor in metastatic groups (Fig. 2G).

**Fig. 3:**
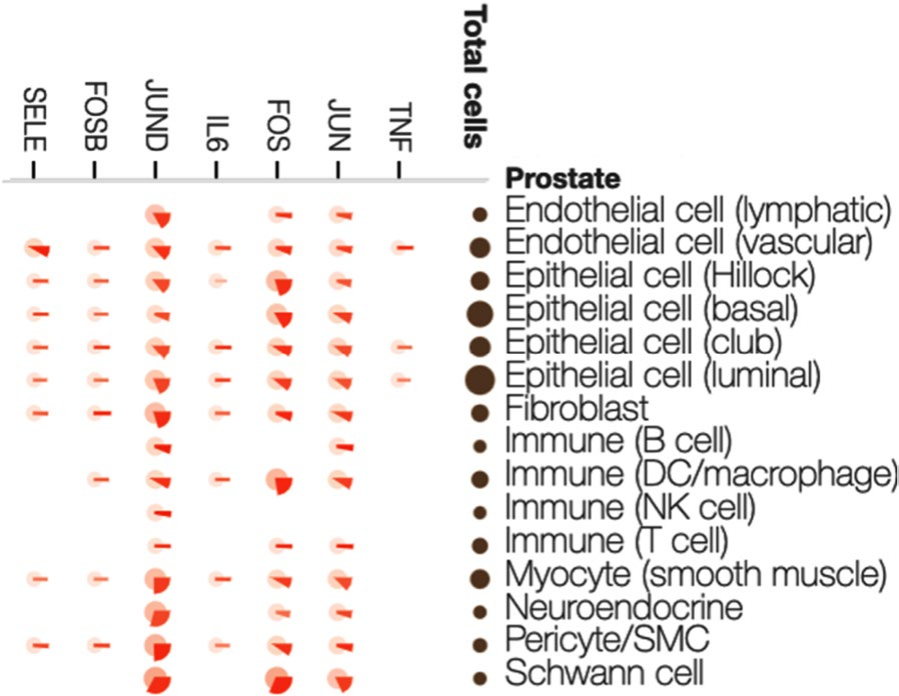
Pie-charts representing the percentage of cells where the expression of a selected gene was detected. The size of each circle in the “Total Cells” column qualitatively represents the proportion of that cell type within the total data

To better assess the significance of TNFα and IL-6 differential expression in PCa progression we analyzed their expression in normal prostate tissue. We accessed the publicly available prostate Genotype-Tissue Expression (GTEx) sc-RNA-seq data for TNFα, IL-6 and AP1 expression (Fig. 3) [28]. GTEx TNFα expression is limited to luminal, club epithelial and vascular endothelial cells but not detectable in immune cells (Fig. 3). However, IL-6 as well as FOSB were expressed in a wider variety of cells including immune cells (Fig. 3).

**Fig. 4:**
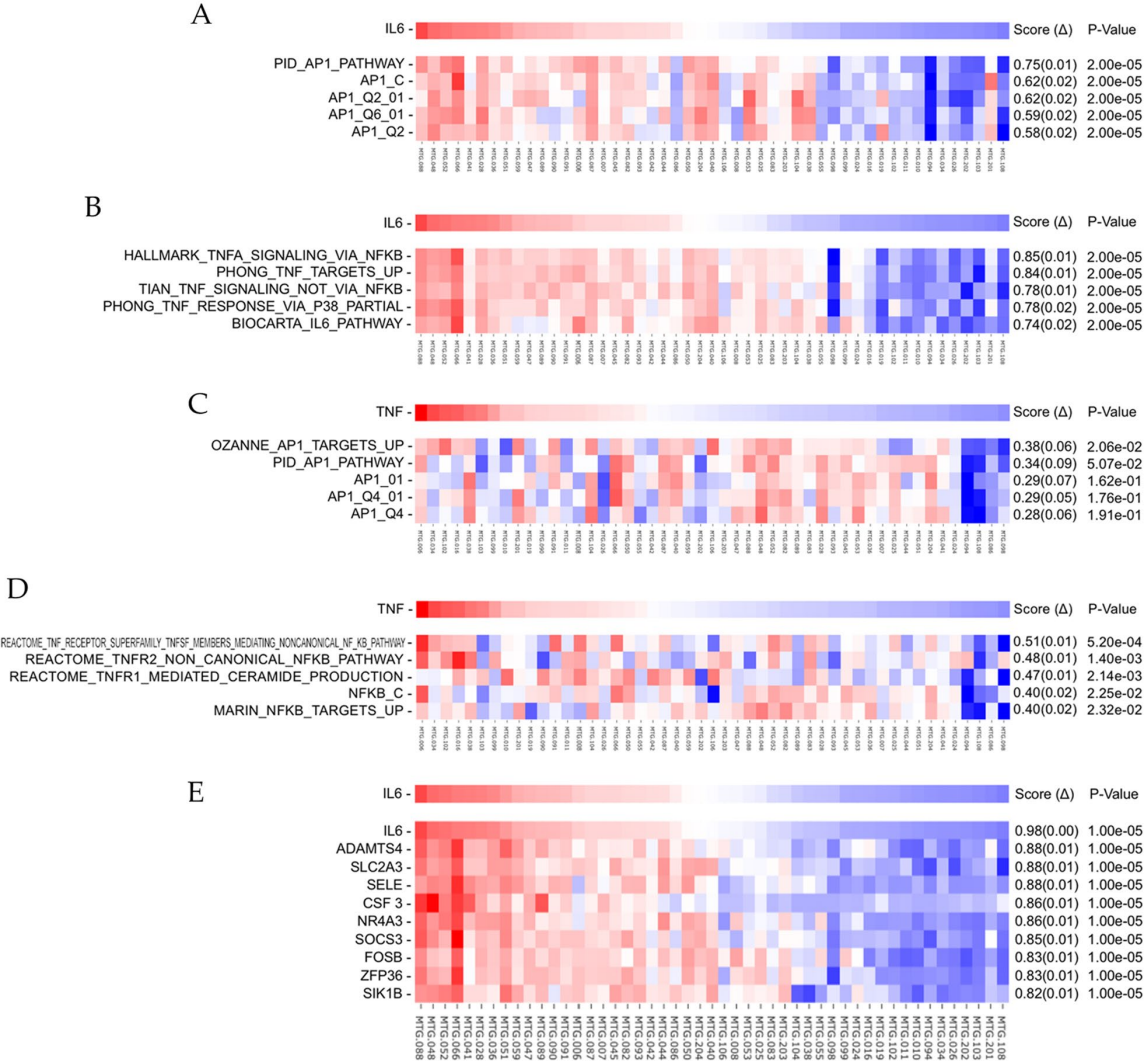
**A–D** Single-sample GSEA analysis heatmaps showing the association between IL-6 and TNFα and the expression of and AP-1 and NF-kB related gene sets. The top 5 scoring gene set are shown **E**. Heatmap showing positive correlation between the relative expression (mRNA) of selected genes, versus the profile of IL-6. The information coefficient (Δ) and associated p-values are shown on the right side of the heatmap. The top 10 scoring genes are shown

We established that TNFα and IL-6 expression in PCa appears to diverge from that of normal prostate tissue, therefore we explored the regulatory signaling pathways implicated in their function. To that end we performed ssGSEA analysis [8], where we projected the patient mRNA profiles onto the space of MSigDB [9, 10] gene sets and matched the ssGSEA profiles against mRNA expression profiles of TNFα and IL-6 using as measure of association the IC (see “Methods”). When matching gene set profiles against IL-6 (mRNA), we observed among the top scoring gene sets those representing AP-1 and NFKB signaling pathways implicated in TNFα regulation. The top scoring gene sets include a gene set representing the AP-1 transcription factor network (PID_AP1_PATHWAY, Fig. 4A) and the MSigDB hallmark that represents genes regulated by NF-kB in response to TNFα (HALLMARK_TNFΑ_SIGNALING_VIA_NFKB, Fig. 4B). However, the top scoring gene sets for TNFα (mRNA) produced a gene set representing cancer motility and invasion genes up-regulated by the AP-1 transcription factor (Ozanne_AP1_TARGETS_UP, Fig. 4C), and one representing TNF receptor superfamily (TNFSF) members mediating the non-canonical NF-kB pathway (REACTOME_TNF_RECEPTOR_SUPERFAMILY_TNFSF_MEMBERS_MEDIATING_NONCANONICAL_NF_KB_PATHWAY, Fig. 4D). As expected, the top scoring ssGSEA results for IL-6 expression are also aligned with localized disease (Fig. 1G, H). They differed for the NFKB pathway where ssGSEA found association with PCa disease progression (TIAN_TNF_SIGNALING_NOT_VIA_NFKB, Fig. 1G).

### E-Selectin is downregulated in PCa metastatic disease

**Fig. 5:**
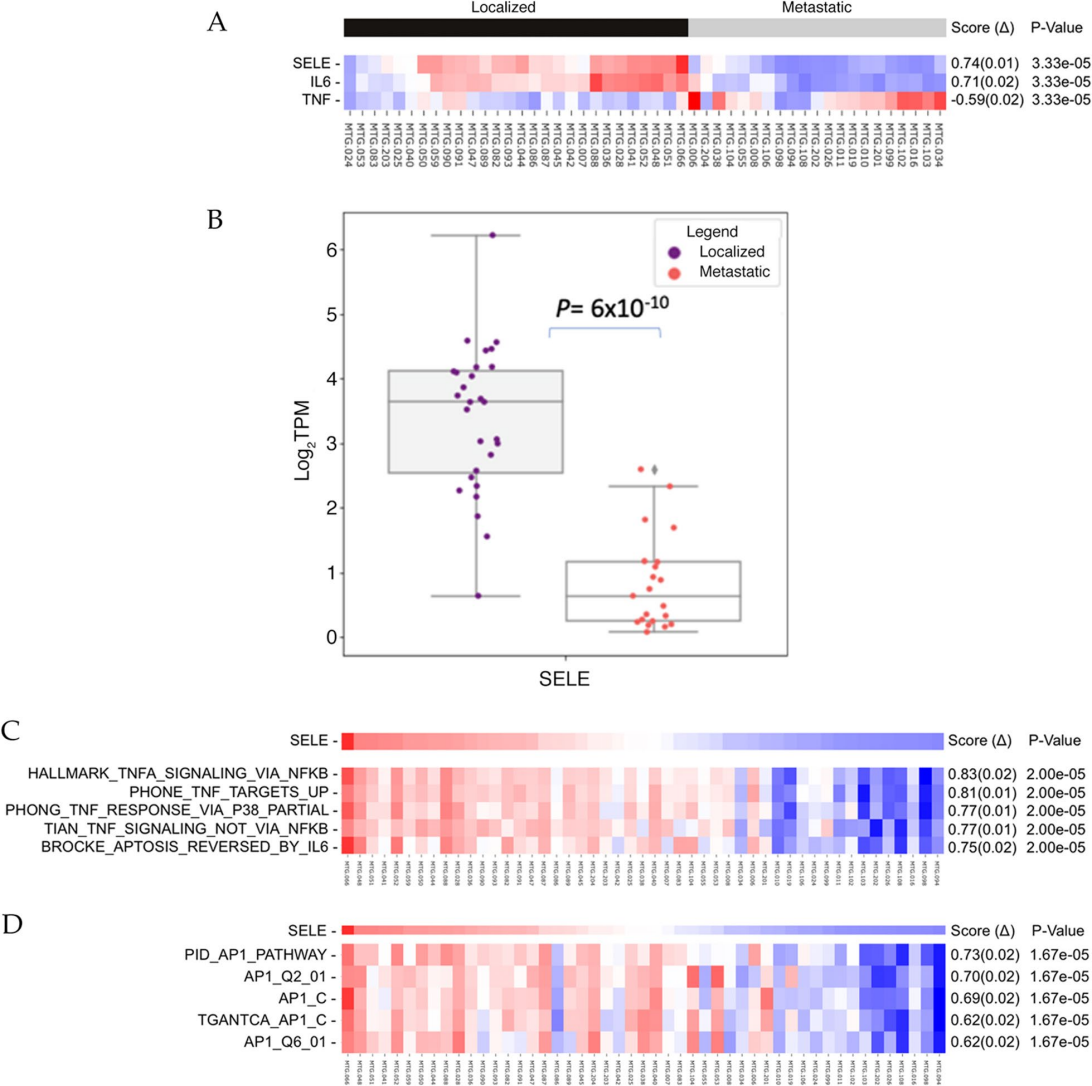
**A** Heatmap showing relative expression profiles (mRNA) of TNFα, IL-6, and SELE vs localized/metastatic groupings of 49 PCa patients, where 28 were localized (black) and 21 were metastatic (grey). **B** Log_2_TPM expression of SELE cohort split by localized and metastatic status. Median and interquartile ranges are shown. **C**, **D** Single-sample GSEA analysis heatmap showing **C** NF-kB related gene sets and **D** AP-1 related gene sets vs. the relative expression (mRNA) of SELE

To identify other factors possibly associated with IL-6 mediated effects on the tumor immune microenvironment (TME), we analyzed the top differentially expressed genes between localized vs. metastatic disease and evaluated their correlation with IL-6. We found 9 genes that were differentially expressed and correlated with IL-6 levels across samples (Fig. 4E). Among these, 2 had previously been implicated in modulation of immune activities: e-selectin (SELE) and ADAMTS4. SELE is implicated in recruitment of leukocytes and is associated with inflammation [29]. It can mediate adhesion of tumor cells to endothelial cells to promote cancer metastasis [30]. We analyzed SELE expression in our patient cohort (Fig. 5A) and observed significant downregulation of this gene in metastatic disease (localized mean = 3.381, metastatic mean = 0.881, p = 6 × 10^–10^, Fig. 5B).

We performed ssGSEA analysis for SELE, and found similar top scoring gene sets as in the results of IL-6, including the AP-1 favored PID_AP1_PATHWAY and NFKB was most associated with HINATA_NFKB_TARGETS_FIBROBLAST_UP (Fig. 5C, D).

**Fig. 6:**
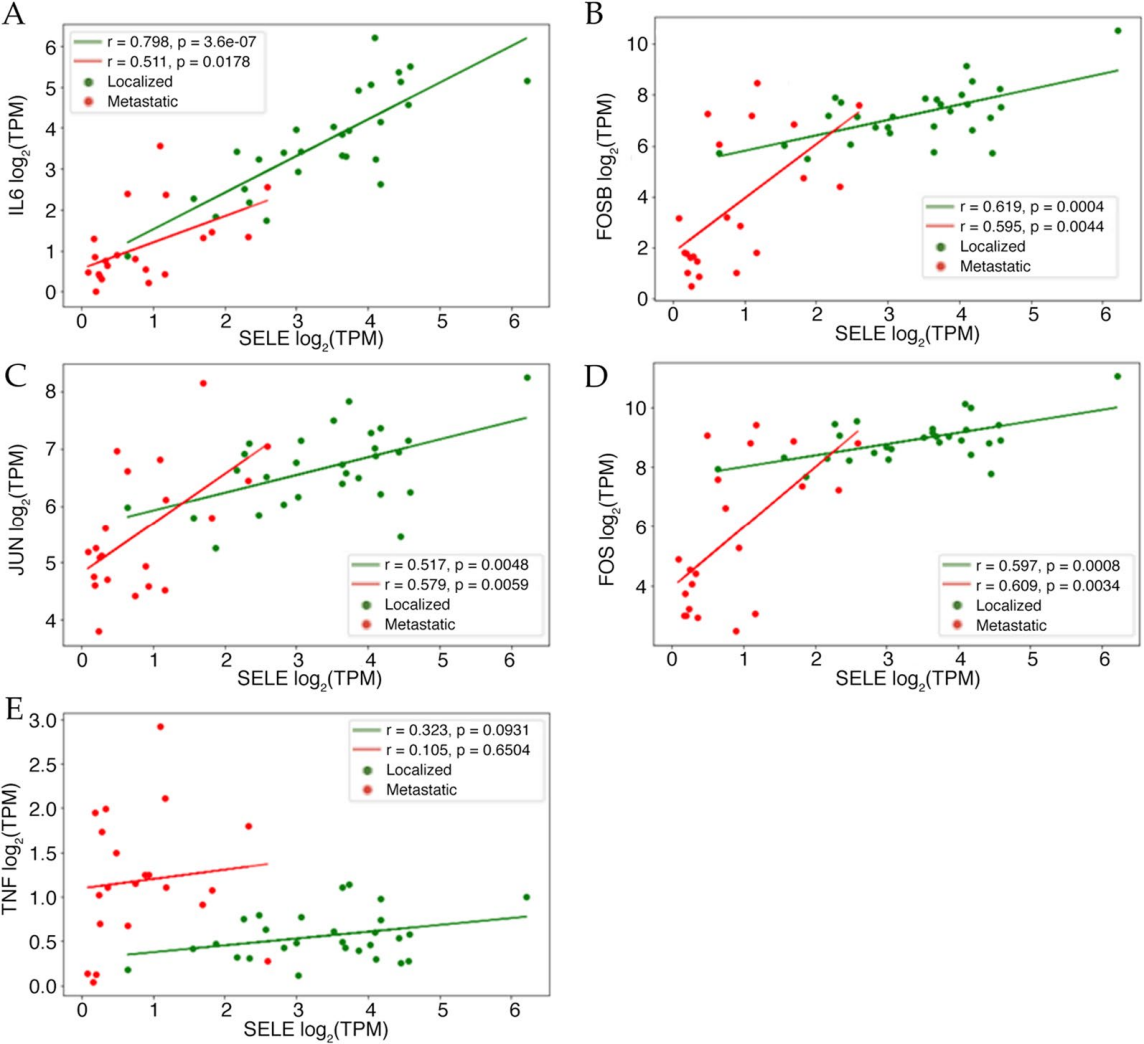
**A**–**E** Scatterplots with Pearson’s R calculated between SELE, IL-6, TNFα, and AP-1 log_2_TPM expression values from RNA-seq analysis of 49 PCa patients. Figure legend keys are as follow: “r”: Pearson’s R; “p”: P-Value; “Localized”: N = 28 localized patients; “Metastatic”: N = 21 metastatic patients; “TPM”: transcripts per million

We performed a Pearson correlation analysis to test the association of SELE expression with AP-1, TNFα and IL-6 (Fig. 6). We observed a strong linear correlation between SELE with IL-6 in both localized and metastatic states, however the correlation is stronger in the localized state (Fig. 6A). FOSB, JUN, and FOS also show a significant correlation with SELE expression (Fig. 6B–D) which is not surprising since AP-1 constitutes part of the SELE promoter.

### PCa immune microenvironment

The PCa immune microenvironment while considered “cold” due to its limited response to immunotherapy has not been fully elucidated. The pro-inflammatory cytokines TNFα and IL-6 as well as SELE are central to immune response regulation and have been implicated as potential targets for PCa therapy. IL-6 antagonists were tested in a clinical setting but failed due to lack of efficacy [6]. There is an ongoing drug development effort in PCa using *uproleselan* (GMI-1271), a SELE antagonist [31]. By analyzing patient bulk RNA-seq data we observed that IL-6 and SELE expression are downregulated in metastatic disease, thus possibly contributing to an immunosuppressive effect (Figs. 4E, 5A). However, TNFα expression increases with disease progression suggesting that regulatory T cells are not engaged in maintaining immune homeostasis to suppress excessive immune responses. Interestingly, whereas we observed enhanced TNFα expression in the metastatic setting, this was associated with downregulation of classical TNFα signaling by ssGSEA analysis (Fig. 1G). This result indicates that TNFα may act in the metastatic setting through non-canonical pathways or on a wider cadre of target cells than previously recognized.

**Fig. 7:**
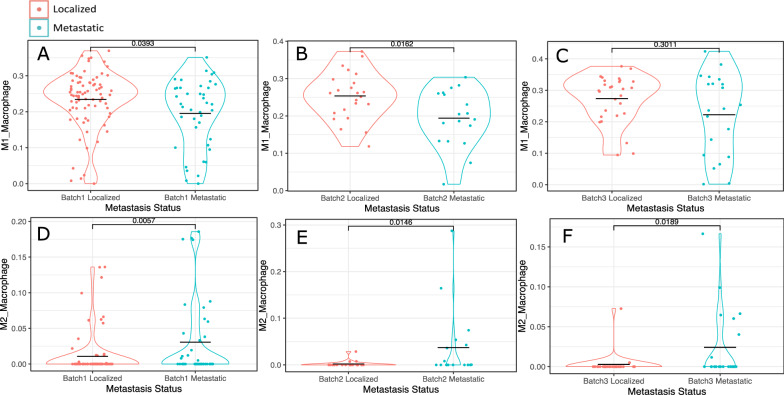
Comparisons of M1 (**A–C**) and M2 (**D–F**) macrophage infiltration of metastatic vs. pre-metastatic patients in batch 1 (**A**, **D**)**,** batch 2 (**B**, **E**), and batch 3 (**C**, **F**). Infiltration estimates were generated using a custom gene signature matrix derived from an annotated single cell RNA-seq dataset of localized and metastatic PCa samples

**Fig. 8:**
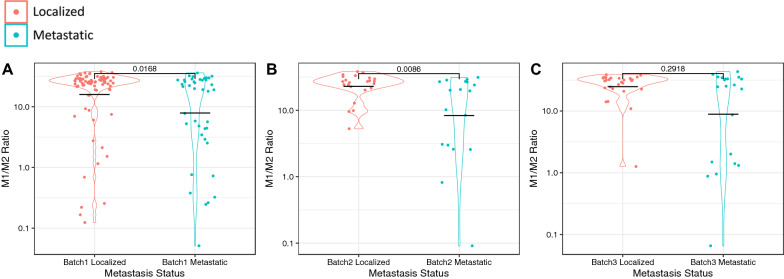
M1 to M2 macrophage infiltration ratio estimates across each cohort, split by localized vs metastatic status. M1 and M2 infiltration estimates were subjected to a pseudocount to avoid infinite ratios. **A** The batch 1 study cohort (n = 120) consisted of 79 localized and 49 metastatic PCa samples. **B** The batch 2 study cohort (n = 41) consisted of 23 localized and 18 metastatic PCa samples. **C** The batch 3 cohort (n = 49) consisted of 28 localized and 21 metastatic PCa samples

**Fig. 9:**
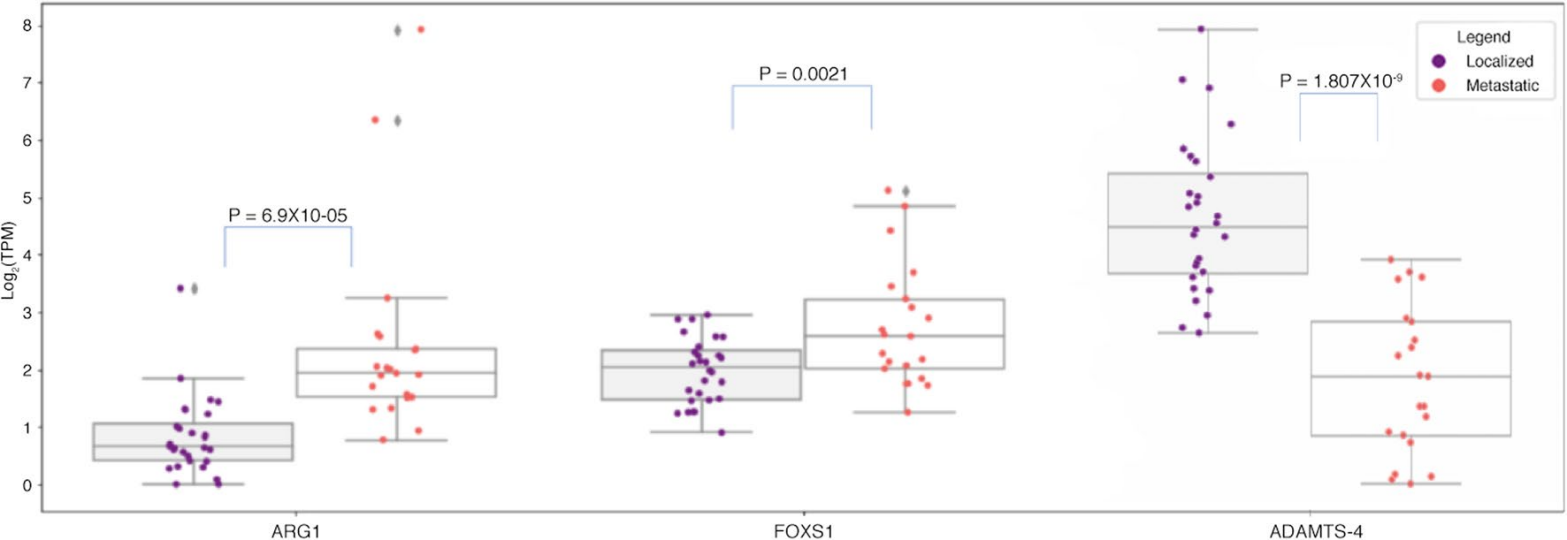
Log_2_TPM expression of ARG1, FOXS1, and ADAMTS-4 in PCa cohort split by localized and metastatic status. These genes were measured to have significantly differential expression between the localized and metastatic groups (two-tailed student’s t-test). Median and interquartile ranges are shown below for each group

Importantly, there is experimental and clinical evidence demonstrating that the pro-inflammatory effect of TNFα can switch to an immunosuppressive function after prolonged exposure [32–34]. To assess the composition of the infiltrating immune cells types, we generated immune cell infiltration estimates using a custom gene signature matrix derived from an annotated sc-RNA-seq dataset of localized and metastatic PCa samples (Table 1). While we found a wide range of infiltrating immune cells, the most significant differences were found for M1 and M2 macrophages (Table 1). Localized disease generally had more M1 macrophages and fewer M2 macrophages than metastatic disease (Table 1, Fig. 7A). To validate this result, we accessed additional RNA-seq patient datasets and found that M2 macrophage enrichment in metastatic disease was reproducible in all our study cohorts (Fig. 7D–F) while the M1 result reproduced in one additional cohort and trended in the other (Fig. 7A–C). Metastatic tumors more often showed reduced M1/M2 macrophage ratios than localized tumors (Fig. 8) and expressed higher levels of ARG1 and FOXS1 which are both associated with M2 macrophage polarization (Fig. 9) [35–38].

**Table 1:** Infiltration estimates in metastatic vs. pre-metastatic samples across all cohorts

All Cell Type Infiltration Estimates									
	BATCH 1			BATCH 2			BATCH 3		
Cell	Pval	Localized Mean	Metastatic Mean	Pval	Localized Mean	Metastatic Mean	Pval	Localized Mean	Metastatic Mean
CD4_Treg	0.5993	0.0074	0.0078	**0.0349**	0.0009	0.0071	0.7618	0.0020	0.0192
CD4_Memory_activated	0.1607	0.1062	0.1147	0.9896	0.1387	0.1270	0.6205	0.0743	0.1032
CD8_Tcell	0.2451	0.0531	0.0421	0.1656	0.0574	0.1026	0.0666	0.0375	0.0641
NKT	0.1366	0.0309	0.0349	**0.0139**	0.0369	0.0475	**0.0289**	0.0266	0.0350
Plasma_Cells	0.3206	0.0530	0.0466	0.6358	0.0533	0.0622	0.9597	0.0372	0.0383
CD8_Tcell_GammaDelta	0.2650	0.0080	0.0083	0.4291	0.0042	0.0018	**0.0076**	0.0345	0.0089
Tcell_follicular_helper	0.2091	0.1206	0.1152	0.0854	0.1403	0.1160	0.1043	0.0988	0.0860
Memory_B_cell	0.9270	0.0053	0.0083	0.3784	0.0367	0.0472	0.8109	0.0029	0.0094
CD4_naive	0.8397	0.0305	0.0314	0.7174	0.0148	0.0154	**0.0441**	0.0510	0.0298
Naive_B_Cell	0.0757	0.0665	0.0737	0.4079	0.0402	0.0350	0.1000	0.0725	0.0924
M0_Macrophage	**0.0370**	0.1158	0.1384	0.6492	0.0882	0.0857	0.0770	0.1115	0.1377
Dendritic_cells_resting	0.9709	0.0020	0.0010	0.3646	0.0009	0.0044	0.1051	0.0000	0.0008
Dendritic_cells_activated	0.2340	0.0089	0.0137	0.2952	0.0019	0.0019	0.9831	0.0163	0.0225
M2_Macrophage	**0.0057**	0.0108	0.0306	**0.0146**	0.0019	0.0371	**0.0189**	0.0028	0.0242
M1_Macrophage	**0.0393**	0.2342	0.1953	**0.0162**	0.2533	0.1943	0.3011	0.2738	0.2224
Monocytes	0.8895	0.0007	0.0068	0.3143	0.0009	0.0007	**0.0234**	0.0069	0.0026
Mast_Cells	0.0555	0.1460	0.1311	0.1537	0.1295	0.1141	**0.0066**	0.1334	0.1033

Wilcoxon ranked sign tests were used to generate p-values. P-values of less than 0.05 are in bold

### Immune sequela of extracellular matrix (ECM) remodeling

Among the genes highly correlated with IL-6 expression, ADAMTS-4 showed the highest association (Fig. 4E). ADAMTS-4, a member of the ADAMTS family of metalloproteinases, was highly expressed in localized samples compared to metastatic disease (localized mean = 4.83, metastatic mean = 2.31, p = 1.8 × 10^–9^, Fig. 9). A major substrate of ADAMTS-4 is the large aggregating extracellular matrix (ECM) proteoglycan versican (VCAN) [39]. VCAN has major functions in tumor cell growth and metastasis, and in our analysis it is expressed robustly both in localized disease and in metastatic samples, underscoring its likely importance along the entire tumor natural history. From the immune perspective, VCAN has been credited with immunoregulatory functions: it acts through Toll-like receptor 2 (TLR2) to dampen antigen presentation by tumor-infiltrating dendritic cells [40]. However, an N-terminal proteolytic fragment of VCAN, versikine, arising through the actions of ADAMTS-4 and other versicanases, conversely regulates cross-presenting type 1 conventional dendritic cell (cDC1) abundance and activation in the TME, to promote immune cell trafficking to the tumor and effector priming [41]. Our data suggest that ADAMTS-4 may contribute to the immune activity in localized tumors through VCAN proteolysis and cDC1 regulation. The increased expression of the master lineage regulator of cDC1, Batf3, as well markers of immune infiltration such as CXCR3 in localized disease, are in agreement with this hypothesis (Fig. 1C). During metastatic progression, attenuated expression of ADAMTS-4, e.g., through TGFb [42], dampens the moderating effects of versikine on the immunoregulatory activities of non-proteolyzed parental VCAN.

### Anti-TNF effect in PCa patients

We demonstrated that TNFα is expressed in PCa throughout disease progression and this pattern could be associated with immune remodeling. TNFα chronic expression is a hallmark of autoimmune disease (AI) widely treated with TNF antagonists. Studies have shown men with AI have a higher risk of all urologic cancers, including bladder, prostate, and kidney cancers [43], and higher incidence of PCa than those without AI diseases [44]. Thus we conjectured that administration of a TNF antagonist may confer therapeutic benefit in PCa. We performed a VHA patient registry study to determine the clinical characteristics associated with anti-TNF therapeutic outcomes. Specifically, we investigated the associations of TNF antagonist administration prior to PCa diagnosis. Our study cohort included 120,204 PCa patients from VHA CDW, among them 390 had TNF antagonist therapy prior to PCa diagnosis. We binarized our cohort into TNF naïve patients (group 1, n = 119,814) and those who received TNF antagonist therapy prior to being diagnosed with PCa (group 2, n = 390, Table 2). The mean age at diagnosis was higher in group 1 (65.76 years vs. 64.96, p = 0.052, Table 2). Patients in group 2 were significantly more likely to be diagnosed with T1 disease than those in group 1 (73.3% vs. 65.9%, p = 0.002, Table 2). Patients in group 2 were less likely to be African American (White: 82.8% vs 69.2%, African American 14.4% vs 27.3%, p < 0.001, Table 2).

**Table 2:** Demographics and baseline disease characteristics of study population

	No TNF use (Group 1)	TNF use pre PCa diagnosis (Group 2)	p Value
Number	119814	390	
Age at Dx [mean(SD)]	65.76 (8.14)	64.96 (6.85)	0.052
Less than 55	8379 (7.0%)	20 (5.1%)	0.179
55–64	49546 (41.4%)	174 (44.6%)	0.21
65–74	43316 (36.2%)	159 (40.8%)	0.066
75 and Up	18573 (15.5%)	37 (9.5%)	0.001
Race			< 0.001
African American	32655 (27.3%)	56 (14.4%)	
White	82894 (69.2%)	323 (82.8%)	
Other	1318 (1.1%)	6 (1.5%)	
Unknown	2947 (2.5%)	5 (1.3%)	
T Stage 1	78899 (65.9%)	286 (73.3%)	0.002
T Stage 2	36839 (30.7%)	98 (25.1%)	0.019
T Stage 3 or 4	4075 (3.4%)	6 (1.5%)	0.059
Gleason 6	48261 (40.3%)	172 (44.1%)	0.138
Gleason 7	47878 (40.0%)	162 (41.5%)	0.56
Gleason 8 or Higher	23690 (19.8%)	56 (14.4%)	0.009
Mean pre diagnostic PSA (SD)	18.15 (71.59)	8.60 (11.99)	0.011
Median pre diagnostic PSA	6.55	5.5	
PSA over 20 at Dx	12253 (11.4%)	23 (6.3%)	0.003
N Stage 1	1965 (1.6%)	6 (1.5%)	1
M Stage 1	4840 (4.0%)	9 (2.3%)	0.108

*TNF* tumor necrosis factor, *Dx* Diagnosis, *SD* standard deviation, *PSA* prostate specific antigen

When performing logistic regression, prior TNF antagonist use (group 2) was associated with reduced odds of presenting with Gleason 8 or higher scores [Odds Ratio (OR): 0.690, p = 0.011, Table 3] and reduced odds of presenting with T stage 3 or 4 disease (OR: 0.447, p = 0.056). Additionally, group 2 showed reduced odds of having PSA over 20 ng/mL at diagnosis (OR 0.572, p = 0.010, Table 3). When measuring associations with metastatic disease at presentation, TNF antagonist use trended towards an association with reduced metastases at diagnosis, but this was not statistically significant (OR: 0.581, p = 0.108, Table 3).

**Table 3:** Associations between group 2 and disease characteristics at PCa diagnosis from multivariable logistic regression models

Outcome	Gleason 8 or higher at diagnosis		T Stage 3 or 4 at diagnosis		PSA Over 20 at diagnosis		Metastases at diagnosis	
	Odds ratio	p Value	Odds ratio	p Value	Odds ratio	p Value	Odds ratio	p Value
Group 2	0.690	0.011	0.447	0.056	0.572	0.010	0.581	0.108
AA Race	1.03	0.044	0.997	0.95	1.75	< 0.001	1.16	< 0.001
Age > 65 years	1.67	< 0.001	1.42	< 0.001	1.92	< 0.001	1.92	< 0.001
Employed at diagnosis	0.821	< 0.001	0.806	< 0.001	0.677	< 0.001	0.685	< 0.001

*PSA* prostate specific antigen, *AA* African American

**Fig. 10:**
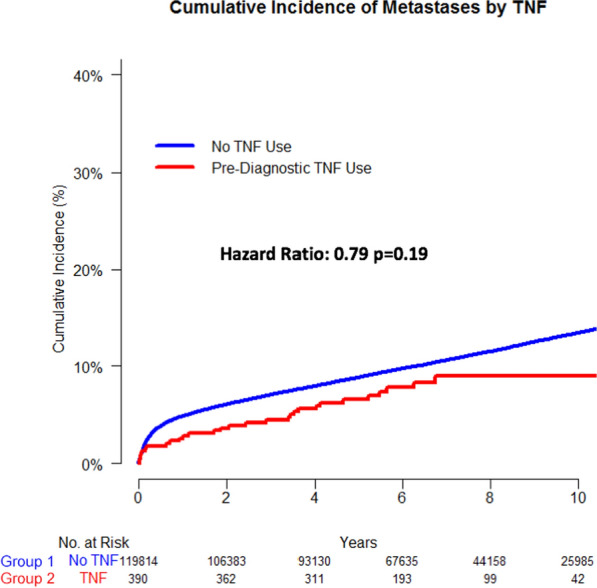
Cumulate incidence of metastases by TNF

Cumulative incidences of metastases at ten years between the two groups were 13.4% for those in group 1 and 8.9% for those in group 2 (p = 0.135, Fig. 10). We applied Cox proportional hazards models and found that group 2 was not associated with long term development of metastases [Hazard Ratio (HR) 0.79, p = 0.19, Fig. 10].

## Discussion

A major obstacle for conducting clinically relevant PCa research has been the lack of cell lines and in vivo experimental models that closely represent human disease progression. To overcome this hurdle, we developed a discovery platform enabling investigation of differential gene expression associated with disease progression.

Our data have implications for understanding the immune and stromal context of PCa progression and metastasis. Whereas both IL-6 and TNFα have pleotropic and stage-specific functions, both cytokines have been implicated in the orchestration of the pre-metastatic niche [45]. Our data highlights an unexpected discrepancy between increased TNFα expression in metastatic samples and reduced enrichment (ssGSEA) of TNFα canonical signatures. This discrepancy may suggest that TNFα promotes metastatic disease through signaling that is distinct from the classical pro-inflammatory pathways triggered by this cytokine.

TFs of AP-1, regulating TNFα, IL-6 and SELE are implicated as oncogenes or tumor suppressors in many cancers [46–48] with drug development programs targeting cJun, JunB, JunD, cFos, FosB, Fra1 and Fra2 [49]. The majority of these studies reported upregulations of AP-1 family members. However, in line with our finding, downregulation of AP-1 TFs has been reported in prostate, gastric, ovarian, colon, cervical and other cancers [49]. Furthermore, downregulation of JUNB/AP-1 in PCa progression was reported by MK Thomsen et al [50]. By focusing on TNFα, IL-6 and SELE function in inflammation we found evidence linking FOSB to PCa disease progression and identified FOSB/AP-1 as a gate keeper. In designing therapeutic intervention targeting FOSB it will be important to address that FOSB function is stage and context specific.

At the level of immune involvement, comparison of the immune cell repertoire between localized and metastatic disease reveals a preponderance of M1 (inflammatory macrophages) in the former transitioning into alternatively activated (M2) macrophages in the latter. This observation suggests that in the primary setting, cancers arising within the physiological structure of the prostate gland are characterized by adaptive immunity that likely favors local growth and propagation of the cancer. Local IL-6-driven inflammation may have direct growth effects on the cancer cells and indirect tumor-promoting effects on the bone marrow microenvironment (e.g., through tolerogenic polarization of antigen-presenting cells). Indeed, earlier work has shown IL-6 to directly promote the growth of prostate carcinoma cells [51]. It is therefore likely that localized cancer arising within the native prostate tissue benefits from sustained local inflammatory networks driven by IL-6, TNFα and SELE. Our data suggest that the inflammatory context radically changes in the setting of non-native metastatic tissue where alternatively activated macrophages pre-dominate. In the metastatic setting, the emphasis shifts from growth promotion (since relatively growth-independent variants have escaped selection and metastasized) to immune evasion, tissue remodeling and angiogenic support. All the latter attributes have been associated in earlier studies with M2 macrophages [52]. Indeed, more recent studies support this hypothesis and show that tumor-associated M2 macrophages, as well as markers of angiogenesis and lymph angiogenesis, predict the prognosis of patients with non-small cell lung cancer [53].

These transitions in immune repertoire between primary and metastatic disease reflect changes in stromal remodeling and its cross talk with anti-cancer immunity. ADAMTS-4, a known target of TGFB immunosuppressive signaling, is downregulated in metastasis. We have previously shown that ADAMTS-4 cleaves the immunomodulatory matrix proteoglycan VCAN. In its intact form, VCAN acts on antigen-presenting cells, dendritic cells and macrophages, to dampen tumor antigen presentation and immune responses. However, a bioactive N-terminal fragment, versikine, arising through the activities of ADAMTS-4 and other versicanases, promotes the abundance and activity of tumor antigen cross-presenting cDC1 subset. Indeed, the relative overexpression of the cDC1 master regulator, Batf3, seems to corroborate this hypothesis. cDC1 are key orchestrators of a “hot” immune microenvironment through chemokine networks that drive T-cell infiltration into the tumor [54].

The increased expression of CXCR3, the receptor for T-cell chemoattractant chemokines CXCL9 and CXCL10 in localized disease appears consistent with enhanced immune milieu of localized specimens. M1 macrophages and immunogenic DC subsets are essential for sufficient local production of CXCL9/10 that drive T-cell-mediated inflammation [55].

In a recent study it was shown that patients with benign prostatic hyperplasia (BPH) and AI who received TNFα antagonists prior to BPH diagnosis were less likely to develop BPH [56]. Importantly, *methotrexate* did not have this effect, further implicating TNFα as a viable target in BPH [56]. Our RNA-seq patient data analysis implicates TNFα as a potential target in PCa. To evaluate the clinical implications of this finding we analyzed the data of 120,204 PCa patients from VHA CDW for outcomes associated with anti-TNF treatment prior to PCa diagnosis. We detected a significant association with earlier grade and stage disease at diagnosis and a trend for improved metastatic propensity.

Taken together, our data demonstrates clear differences in immune contexture between localized and metastatic disease in PCa. Primary localized disease demonstrates features of local inflammation and adaptive immunity, likely counterbalanced by immune checkpoint-driven T cell exhaustion and/or defects in antigen presentation. By contrast, metastases demonstrate immune cold microenvironments and a shift towards resolution of inflammation and tissue repair. Our data provide novel insights into the potential mechanisms accounting for the modest efficacy of immune checkpoint inhibitors in advanced PCa and suggest that combinations of immunotherapy with anti-angiogenic or stroma-modifying therapy may improve patient outcomes. In this context, clinical trials with antiangiogenic agents such as *bevacizumab* yielded conflicting results in the treatment of PCa [57]. However, combinations of *bevacizumab* with immune checkpoint inhibitors in difficult-to-treat tumors such as hepatocellular carcinoma are now standard of care [58]. It is tempting to speculate that such a combination, perhaps in conjunction with anti-TNF therapy, will lead to important therapeutic advances.

## Conclusion

Our data points to clearly different inflammatory contexts between localized and metastatic prostate cancer. Primary localized disease demonstrates local inflammation and adaptive immunity, whereas metastases are characterized by immune cold microenvironments and a shift towards resolution of inflammation and tissue repair. Therapies that interfere with these inflammatory networks may offer opportunities for early intervention in monotherapy or in combination with immunotherapies and anti-angiogenic approaches.

## Data Availability

The datasets used, generated, and analyzed during the current study are available from the corresponding author on reasonable request.

## Abbreviations

AI: Autoimmune disease
AJCC: American Joint Committee on Cancer
AP-1: Activator protein 1
BPH: Benign prostatic hyperplasia
cDC1: Type 1 conventional dendritic cell
CDW: Corporate data warehouse
CISM: Chromogenic in situ hybridization
ECM: Extracellular matrix
GTEx: Genotype-tissue expression
HR: Hazard ratio
IC: Information coefficient
IL-6: Interleukin 6
IRB: Institutional review board
mRNA: Messenger RNA
MSigDB: Molecular signatures database
NGS: Next generation sequencing
NLP: Natural language processing
OR: Odds ratio
PCa: Prostate cancer
q-RT-PCR: Quantitative reverse transcription PCR
RNA-seq: RNA-sequencing
ssGSEA: Single-sample gene set enrichment analysis
TFs: Transcriptional factors
TLR2: Toll-like receptor 2
TME: Tumor microenvironment
TNFα: Tumor necrosis factor alpha
TNFSF: TNF receptor superfamily
VCAN: Versican
VHA: Veterans health administration
VINCI: Veterans affairs informatics and computing infrastructure
